# Introducing the construct of risky cannabis use: designing and piloting a co-created educational intervention on cannabis health literacy among adolescents and young adults. The CAHLY (CAnabis Health LiteracY) study.

**DOI:** 10.1192/j.eurpsy.2024.593

**Published:** 2024-08-27

**Authors:** E. Caballeria, C. Oliveras, P. Guzmán, M. Ballbé, B. Fleur, B. Pol, D. Ilzarbe, H. López-Pelayo, S. Matrai, M. Artigas, M. T. Pons-Cabrera, D. Folch, L. Nuño, M. Balcells-Oliveró

**Affiliations:** ^1^Health and Addictions Research Group (Grup de Recerca Emergent, 2021 SGR 01158, AGAUR), IDIBAPS; ^2^Addictions Unit. Psychiatry and Psychology Service, Hospital Clinic de Barcelona; ^3^Tobacco control Unit, Institut Català d’Oncologia - IDIBELL; ^4^IDIBAPS; ^5^Department of Child and Adolescent Psychiatry and Psychology, SGR-881, Hospital Clinic de Barcelona; ^6^CIBERSAM, Barcelona, Spain

## Abstract

**Introduction:**

Cannabis use poses a significant risk to the psychological wellbeing of youth, affecting academic performance and potentially triggering the onset of mental health issues. Providing young people with comprehensive information about patterns of cannabis use and specific factors that increase an individual’s health risks is crucial. The ability to critically assimilate this information is known as health literacy (HL).

**Objectives:**

To design a psychoeducational intervention to increase HL on risky cannabis use among students aged 16-25, and to assess its usability and feasibility.

**Methods:**

We designed a psychoeducational intervention based on the outcomes of a 3-hour co-creation session involving healthcare professionals and students. 29 university students and 25 high-school students completed this intervention and assessed its usability and feasibility with the SUS (System Usability Scale), PSSUQ (Post-Study System Usability Questionnaire) and additional open questions regarding the most and less-liked aspects of the intervention.

**Results:**

The design phase resulted in an informative website (http://www.cahlyclinic.cat/) and a 1-hour structured onsite educator-facilitated session, comprising 3 group activities (completed on paper or online) addressing three dimensions of cannabis HL: searching for, interpreting and applying reliable information. Usability of the intervention was rated as excellent (SUS mean score>80). PSSUQ results indicate that students were satisfied with the intervention; found the HL information clear, relevant, and adequate for their needs; found the interface of the digital version pleasant and usable without support; and would recommend it to other students.

**Conclusions:**

We propose an innovative structured and usable intervention, designed using a participatory approach, which aims to disseminate information on risky cannabis use to a key target population, namely young people.

**Disclosure of Interest:**

None Declared

